# In vitro delayed response to dihydroartemisinin of malaria parasites infecting sickle cell erythocytes

**DOI:** 10.1186/s12936-023-04819-5

**Published:** 2024-01-04

**Authors:** Albert A. Gnondjui, Offianan A. Toure, Berenger A. Ako, Tossea S. Koui, Stanislas E. Assohoun, Eric A. Gbessi, Landry T. N’Guessan, Karim Tuo, Sylvain Beourou, Serge-Brice Assi, Francis A. Yapo, Ibrahima Sanogo, Ronan Jambou

**Affiliations:** 1 Unité de Paludologie, Institut Pasteur Côte d’Ivoire, 01 BP 490 Abidjan 01, Côte d’Ivoire; 2 Laboratoire Biologie et Santé, Université Felix Houphouët Boigny, Abidjan, Côte d’Ivoire; 3 Laboratoire de Mécanique et Informatique, Université Felix Houphouët BoignyCôte d’Ivoire, Abidjan, Côte d’Ivoire; 4 Institut Pierre Richet/Programme National de Lutte contre le Paludisme, Bouaké, Côte d’Ivoire; 5 Service d’Hématologie, CHU Yopougon, Abidjan, Côte d’Ivoire; 6 Present Address: Global Health Department, Institut Pasteur Paris, 25 rue du Dr Roux, 75015 Paris, France

**Keywords:** Sickle cell anemia, Artemisinin combination therapy, *Plasmodium falciparum*, Anti-malarial drugs, Treatment resistance, In vitro drug monitoring

## Abstract

**Background:**

Decreased efficacy of artemisinin-based combination therapy (ACT) for *Plasmodium falciparum* malaria has been previously reported in patients with sickle cell disease (SCD). The main purpose of this study was to investigate the in vitro susceptibility of isolates to dihydro-artemisinin (DHA) to provide a hypothesis to explain this treatment failure.

**Methods:**

Isolates were collected from patients attending health centres in Abidjan with uncomplicated *P. falciparum* malaria. The haemoglobin type has been identified and in vitro drug sensitivity tests were conducted with the ring stage assay and maturation inhibition assay.

**Results:**

134 isolates were obtained. Parasitaemia and haemoglobin levels at inclusion were lower in patients with haemoglobin HbSS and HbSC than in patients with normal HbAA. After ex vivo RSA and drug inhibition assays, the lowest rate of parasitic growth was found with isolates from HbAS red cells. Conversely, a significantly higher survival rate of parasites ranging from 15 to 34% were observed in isolates from HbSS. Isolates with in vitro reduced DHA sensitivity correlate with lower RBC count and haematocrit and higher parasitaemia at inclusion compared to those with isolates with normal DHA sensitivity. However, this decrease of in vitro sensitivity to DHA was not associated with Kelch 13-Propeller gene polymorphism.

**Conclusion:**

This study highlights an in vitro decreased sensitivity to DHA, for isolates collected from HbSS patients, not related to the Pfkelch13 gene mutations. These results are in line with recent studies pointing out the role of the redox context in the efficacy of the drug. Indeed, SCD red cells harbour a highly different ionic and redox context in comparison with normal red cells. This study offers new insights into the understanding of artemisinin selective pressure on the malaria parasite in the context of haemoglobinopathies in Africa.

## Background

Parasitic infections, such as *Plasmodium falciparum* malaria, are one of the major causes of morbidity and mortality in patients with HbSS, HbSC and HbCC sickle cell phenotypes [1–3]. Indeed, the development of the parasite in sickle cells [4–6], may cause vaso-occlusive crises and increase haemolytic anaemia through acute haemolysis episodes [7, 8]. Malaria is considered to be one of the main causes of hospitalization for patients with sickle cell disease (SCD) [2, 9]. Studies showed that the sickle cell trait (heterozygous HbAS) does not prevent malaria infection but protects against severe malaria [10, 11]. Despite a lower risk of malaria infection, people with homozygous status (HbSS) are at a higher risk of mortality [12, 13].

Resistance to artemisinin-based combination therapy (ACT) emerged in the Mekong region known to have a very high prevalence of haemoglobin E [14–16]. Recent studies showed that resistance of *P. falciparum* to artemisinin and its derivatives was based on a quiescence mechanism during the ring stage of the parasite [17, 18]. In Asia, this resistance was attributed to mutations in the propeller region the Kelch13 gene *k13* [19]. Polymorphisms in the *k13* gene have been recently found in Africa (Y493H, P553L, R561H, M476I, P574L, C580Y and A675V), including Ghana, Rwanda, Uganda, Tanzania [20–23]. Some of the mutations found in African countries are correlated with delayed parasite clearance. However, red blood cells (RBCs) with abnormal haemoglobin differ from normal RBCs in terms of redox potentials and calcium fluxes [24, 25]. They express higher level of reactive oxygen species (ROS) [26] and harbour a defect in antioxidant system [27] associated with externalization of phosphatidylserine [28] associated with capture of the cell by the spleen. ROS and superoxide are also involved in mechanism of action of artemisinin and in the resistance of the parasite [29, 30]. Metabolic changes observed in abnormal red cells could thus induce a high selective pressure on all the ionic regulation pathways of the parasite [4]. They could modulate the metabolic pathways involved in artesunate resistance and selecting resistant parasites, or decreased the efficacy of DHA on its target(s).

In the other side, and despite early description of mutations in the SERCA gene, other genes have been described to correlate with the decrease in artemisinin sensitivity [21, 22]. These genes are ferredoxin (*fd*), apicoplast ribosomal protein S10 (*arps10*), *Plasmodium falciparum* multidrug resistance protein 2 (*pfmdr2*), *Plasmodium falciparum* chloroquine resistance transporter (*Pfcrt*), *Plasmodium falciparum* adaptor protein complex 2 mu subunit (*pfap2mu*) and *Plasmodium falciparum* ubiquitin-specific protease 1 (*pfubp1*) [31–35]. All these genes are involved in the homeostasis of the cell content and their expression could be highly modulated when facing the very particular cytosol content of the abnormal red cells.

In Côte d’Ivoire, the studies by Tossea et al. showed, a prevalence of the major form (HbSS, HbSC) and the minor form (HbAS) of sickle cell disease in patients with uncomplicated malaria of 2% and 6%, respectively [36]. Since 2005, Côte d’Ivoire has been using artemisinin-based combinations in the treatment of uncomplicated *P. falciparum* malaria. A high prevalence of polymorphisms in the *k13*- gene of parasite isolates, was also described [37]. At the same time, Adjei et al*.* [38] in Ghana, as well as Gbessi et al*.* in Côte d’Ivoire [39] reported decreased efficacy of ACT in sickle cell patients correlated with a delay in *P. falciparum* clearance. This suggests a possible resistance of *P. falciparum* after treatment with artemisinin-based combinations in this population. Gbessi et al. [39] highlighted that a larger phenotypic complexity was found in the parasite populations of patients with SCD than in normal ones. However, the in vivo therapeutic efficacy test for ACT does not allow a direct analysis of parasites response to artemisinin derivatives because the additional effect of the second drug and of patient’s immunity on drug efficacy could mask the detection of chemo-resistant isolates in high transmission area.

Development of in vitro studies of *P. falciparum* with low sensitivity to artemisinin derivatives in sickle cell patients are urgently needed to discriminate between mechanisms involved i.e., mutation of genes or transcriptomic regulation gene expressions, and/or partial inactivation of the drug in the cytosol of the red cell. The aim of this study was to investigate the in vitro susceptibility to DHA, of parasites inducing malaria in sickle cell patients. For this purpose, phenotypic tests (ring stage assay and schizont maturation tests) and genotypic test (*k13* gene sequence analysis) were carried out to evaluate the susceptibility of these parasites to DHA.

## Methods

### Ethical considerations

Studies were conducted according to the declaration of Helsinki and national legal and regulatory requirements. Protocol, case report form, and informed consent form were approved by the National Ethics and Research Committee of the republic of Côte d’Ivoire. An informed consent was required from each participant and/or parents or legal guardians of children. For children over the age of 9, informed consent was required prior to their inclusion in the study.

### Study sites

The study was conducted from May 2017 to February 2020 in Côte d'Ivoire (RCI). In RCI and its neighbouring countries (Ghana, Burkina Faso and Mali) a high rate of sickle cell disease is found with a prevalence between 4 and 25% of the genetic traits [40, 41]. Due to its strategic geographical position between the Golf of Guinea and the Sahel, it is subject to a high migratory flow. Thus, crossbreeding and consanguineous marriages in Côte d'Ivoire are responsible for a sickle cell trait (HbAS, HbAC, HbSS and HbSC) rate of around 14%, with 2% of HbSS and HbSC phenotypes [42, 43]. Data obtained during clinical trials on the efficacy and tolerance of ACT conducted in different regions in CI, highlighted a prevalence of sickle cell disease in malaria patients around 2% for HbSS and HbSC phenotypes and 6% for the sickle cell trait (HbAS) [36].

This prospective study was carried out at the Clinical Hematology department at Yopougon University Hospital (YUH, Abidjan) and at the community health centre of Anonkoua-kouté (ANK, Abidjan). YUH is the reference centre for sickle cell disease in Côte d’Ivoire where about 10,000 patients with HbSS, HbSC and HbCC phenotypes are followed up with free access to medical care. ANK is a secondary level health structure which receives more than 400 patients daily. Patients attending this health centre can benefit from the typing of haemoglobin using acid acetate electrophoresis.

### Patients recruited and samples collection

For patients suffering from fever attending both health structures, a clinical examination was performed before a biological confirmation of malaria. A first screening of malaria was carried out by lateral flow test. Positive results were validated by examination of thick and thin blood Giemsa-stained smears at × 100 with light microscopy. For *P. falciparum* positive sample, parasite density was calculated. After written informed consent of participants or of their legal representatives, all patients over 6 months of age with a parasite density beyond or equal to 0.1% were included in the study. An electrophoresis of haemoglobin was performed to all the patients registered. For enroled patients, a questionnaire was applied including demographic data, sex, age, place of residence, body temperature and clinical symptoms.

Patients with signs of severe malaria (WHO criteria) and/or requiring intensive medical care for other severe diseases, as well as those already treated with antimalarial drugs or antibiotics within the 30 days prior to medical consultation were not include and directly addressed to physician consultation with their biological results.

In YUH only patients with already known SCD and malaria were recruited. Whereas in Anonkoua-Kouté health centre all the patients with positive thick blood smears for *P. falciparum* were enroled after informed consent notwithstanding the result of the electrophoresis.

For each patient 3 mL of peripheral venous blood were collected on EDTA tubes for culture, and 2 ml of blood were collected on dry tubes for biochemical tests (CRP). Blood spots were also done with three drops (50 µl each) put on a Whatmann 3MM® filter paper and dried at room temperature for 4 h. For the two sites, samples were kept at 4 °C in an ice chest cooler and sent to the Malaria Unit of the Institut Pasteur of Côte d’Ivoire in less than four hours.

### Haemoglobin status

Patients enroled at YUH were already aware of their sickle cell status and were all carriers of major forms (HbSS, HbSC and HbCC). These patients were routinely treated and followed up by the reference centre. Nevertheless, they genetic status was confirmed by PCR/ FRET (Fluorescence Resonance Energy Transfer) method [44]. At the Anonkoua-Kouté Health Centre, screening for SCD diagnosis was done by electrophoretic using an SAIO Electrophoresis instrument (PSE; Italy).

### In vitro drug sensitivity test

In vitro tests were performed using RPMI-1640 (Eurobio 479604, 500 ml) medium supplemented with 5% Albumax II, 1% L-glutamine, 2% D-glucose, 0.05% hypoxanthine, 2.5% HEPES (Eurobio 251010) buffer and 0.5% gentamicin (Eurobio 524221). Serum and buffy coat were removed from the whole blood obtained from patients and red blood cells were washed three times in RPMI-1640 medium (centrifugation at 3000 rpm for 10 min) prior to cultivation. Samples were seeded in culture less than 5 h after blood collection. The cultures were conducted in a modular incubator chamber saturated with 5% O_2_, 5% CO_2_ and 90% N_2_ in a humidified atmosphere.

### Ring-stage survival assay

The ex vivo RSA test was conducted according to Witkowski et al*.* [45] with minor modifications. To confirm viability of clinical isolates, two concentrations of dihydro-artemisinin (DHA) were used for each isolate, i.e. 700 nM and 70 nM. Dimethylsulfoxide (DMSO) at 0.1% was used as negative control.

The rest of the procedure did not change. Parasite culture mixture adjusted to 2% haematocrit was prepared. Initial parasitaemia of the isolates was between 0.1 and 1% and no new uninfected RBCs was added. Also, isolates were not synchronized prior to the assay. Briefly the modular incubator chamber was placed in an incubator at 37 °C for six hours. After 6 h of exposure to DHA, the red blood cells were washed three times with a preheated RPMI 1640 medium and suspended in a new complete medium. Cultures were incubated under the same conditions for sixty-six hours. At the end of the culture period (i.e., 72 h), Giemsa-stained thin blood smears were prepared and examined. The number of infected red blood cells containing viable parasites was counted by two independent investigators in a total of at least 10,000 red blood cells. Viable parasites with normal morphologic appearance (either ring stages, trophozoites, or schizonts) were counted to determine the survival rate [45]. The tests were considered to be valid when the parasitaemia at 72 h, in wells without any DHA (nonexposed culture) was higher than the initial parasitaemia [45]. Survival rates were calculated as the ratios of parasitaemia in wells with DHA (exposed) and in wells without (nonexposed) [45]. Parasite isolates demonstrating a survival rate higher than 1% in the RSA were considered to display reduced susceptibility to artemisinin [45, 46]. Based on previous comparative studies of ex vivo RSA and in vivo drug susceptibility tests [45, 46], a high survival rate higher than 10% is likely to have a clearance half-life after artemisinin treatment higher than five hours (cut-off with 89% sensitivity and 91% specificity).To confirm adequate culture conditions, the RSA tests were performed with two *P. falciparum* reference strains, i.e.K1 (artemisinin sensitive strain) and IPC 3445 (Cambodian strain resistant to artemisinin) as negative and positive control, respectively.

### Maturation inhibition assay

The in vitro *P. falciparum* maturation test was conducted as developed by Jensen and Trager [47], standardized by Le Bras and Durand [48] and modified for fluorescent detection by Smilkstein et al. [49] and Basco [50]. Parasitized red blood cells were seeded in complete medium at haematocrit of 2%. In case of parasitaemia greater than 0.3%, type O positive washed healthy human erythrocytes were added to adjust parasitaemia to 0.3%. Dihydroartemisinin was added in duplicate in 96-well microtiter plate at concentrations ranging from 35.16 nM to 0.55 nM. As previously, incubation was conducted at 37 °C for 72 h in a modular incubator chamber saturated with 5% O_2_, 5% CO_2_ and 90% N_2_ in a humidified atmosphere. After incubation, cultures were frozen for 24 h to stop parasite growth. Cultures were thawed and parasite growth was assessed by SYBR Green I incorporation method using a spectrofluorometer (DELL, FLx800, Biotek) according to Smilkstein et al*.* [49], Basco [50] and Le Nagard et al*.* [51]. Drug concentrations inhibiting 50% of the parasite growth (IC_50_) were determined using IVART (In vitro Analysis and Reporting Tool) software from WWARN's [52]. Validation of each test was also assessed with IVART. Resistance thresholds of DHA (10 nM) was defined according to IVART. As a reference, K1 artemisinin-susceptible strain and MRA-1236 artemisinin-resistant clone (IPC 3445) were used. They were provided by the Malaria Research and Reference Reagent Resource Centre [53].

### k13-propeller gene sequencing

Parasitic DNA was extracted from dried blood spots using a Qiagen kit according to the manufacturer's instructions. The fragment 1279–2127 of the coding sequence of *k13* gene of *P. falciparum* was amplified by nested PCR according to Ariey et al. [19]. PCR products were sequenced according to Sanger method by Genewiz compagny. Sequences aligned by Seaview 5 were analysed using BioEdit software version 7.0.9.1 and compared to the *k13* sequence (XM_001350122.1).

### Statistical analysis

Statistical tests were performed using GraphPad Prism 7.0 version (GraphPad prism software Inc., San Diego, CA, USA), and Statistica v9. The Shapiro–wilk test was used to verify data normality. Medians with interquartile deviations were used for data that do not follow a normal distribution. Mann–Whitney U-test, Kruskal Wallis test and median test were used to compare groups. Correlations were determined using the Spearman test or Kendall Tau test. Comparisons were considered statistically significant when p ≤ 0.05.

## Results

### Study population

**Fig. 1:**
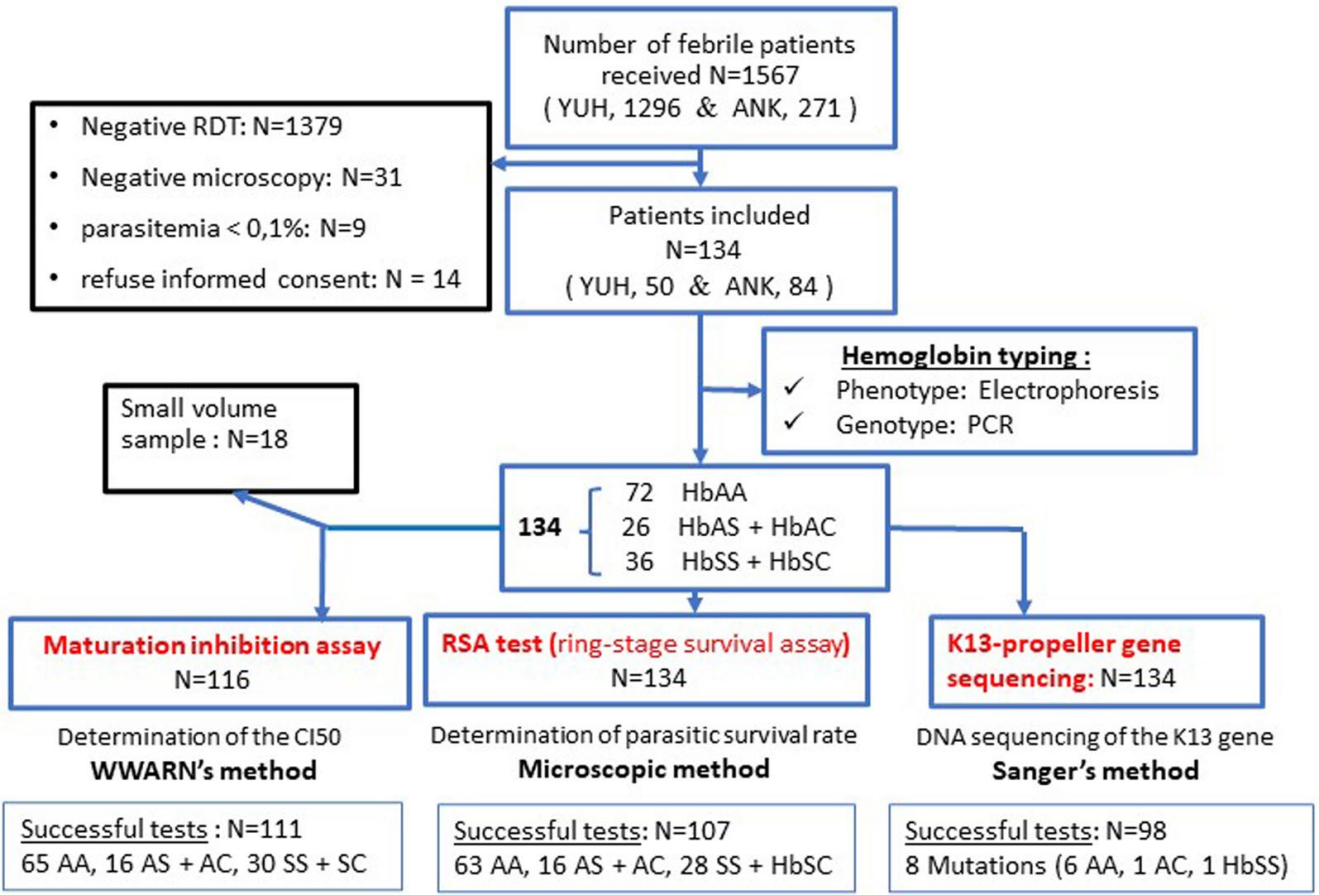
Flow chart of the study

During the study, a total of 1567 patients attended health centres with suspected mild malaria infection. However, most of them (Fig. 1) had either a negative rapid test or a negative thin blood smear or have already undergone treatment, and were not included in the study. A total of 134 patients were enroled and blood sampled. Among them 72 had HbAA phenotypes, 26 were heterozygotes (HbAC or HbAS) and 36 were double mutated (HbSC or HbSS)** (**Fig. 1). Surprisingly no HbCC was found.

In the present study, 70% of the recruited patients were children with ages ranging from 1 to 14 years old (Table 1). Also, there is no age difference between children with normal haemoglobin HbAA, pathological haemoglobin (HbSS, HbSC) or asymptomatic form (HbAS

 and HbAC) in (Tables 1 and 2). Patients had usually received non-steroidal anti-inflammatory drugs before attending health centres. 81% (29/36) of the participants with a mutated haemoglobin (HbSS and HbSC). The mean temperature and median parasite density at inclusion were lower in patients with HbAS, HbSC and HbSS compared to normal HbAA group (Mann Whitney test, P < 0.001). Likewise, erythrocyte count, haemoglobin and haematocrit were lower at inclusion for HbAS, HbSC and HbSS patients compared to HbAA (Mann Whitney test, P < 0.001). This difference was also found between HbAC and HbSC groups (Mann Whitney test, P < 0.005), and HbSS versus HbAS groups (Mann Whitney test, P < 0.0003) (Table 1). Patients with abnormal HbSS phenotypes had lower haematological parameters than those with HbAS and HbSC forms. Indeed, anaemia (Hb < 11 g/dl blood) was more often found in patients with sickle cell phenotypes HbAS, HbSC and HbSS, than in HbAA (11.28 ± 2.10 g/L) and was more severe for HbSS phenotypes (6.51 ± 2.24 g/L) (Mann Whitney test, p < 0.001).

**Table 1:** Basic parameters of the study population

	HbAA (n = 72)	HbAC (n = 7)	HbAS (n = 19)	HbSC (n = 13)	HbSS (n = 23)
Sexe ratio (M/F)	1.1	2.5	0.4	0.5	1.6
Age (years), (Mean ± SD)	12.90 ± 11.08	13.47 ± 10.67	12.43 ± 4.76	10.17 ± 4.42	19.15 ± 14.12
Body mass index (mean ± SD)	20.65 ± 5.77	21.67 ± 4.54	19.06 ± 5.40	18.75 ± 4.22	14.41 ± 3.41
Temperature (°C), (mean ± SD)	39.58 ± 1.16	39.87 ± 1.56	38.29 ± 0.92	38.28 ± 0.71	38.22 ± 1 .13
Parasitaemia (/µL of blood), Median (IQR)	38315 (19515—53543)	11200 (5610—57100)	13670 (8220–42600)	13,250 (4805–21800)	16,300 (7900—43900)
Leukocytes (× 1000/µL), (mean ± SD)	17.57 ± 15.71	13.51 ± 13.07	16.80 ± 9.82	23.58 ± 22.68	17.83 ± 7.80
Erythrocytes (million/µL), (mean ± SD)	4.65 ± 1.00	4.35 ± 0.99	3.44 ± 1.75	4.03 ± 1.79	2.48 ± 1.44
Platelets (× 1000/µL), (mean ± SD)	233 ± 78.63	205.20 ± 88.80	282.10 ± 106.21	189.37 ± 79.92	261.47 ± 114.75
Haemoglobin level (G/100mL), (mean ± SD)	11.28 ± 2.10	13.05 ± 2.53	8.66 ± 2.38	9.63 ± 1.71	6.51 ± 2.24
CRP, (mean ± SD)	55.31 ± 50.29	65.86 ± 61.84	68.74 ± 52.39	66.31 ± 46.51	68. 35 ± 40.60
Haematocrit (%), (mean ± SD)	38.64 ± 4. 60	43.55 ± 8.97	26.21 ± 7.88	27.15 ± 6.11	18.66 ± 6.71

*p-Values*	HbAA Vs HbAC	HbAA Vs HbAS	HbAA Vs HbSC	HbAA Vs HbSS
Age (years)	0.383	0.561	0.067	0.728
Body mass index	0.677	0.359	0.262	** < 0.001**
Temperature (°C)	0.678	** < 0.001**	** < 0.001**	** < 0.001**
Parasitaemia ( /µL of blood)	0.061	**0.012**	** < 0.001**	**0.014**
Leukocytes (× 1000 / µL)	0.616	** < 0.001**	0.248	**0.054**
Erythrocytes (million/µL)	0.576	** < 0.001**	** < 0.001**	** < 0.001**
Platelets (× 1000/µL)	0.228	0.086	0.218	0.277
Haemoglobin level (G/100mL	0.083	** < 0.001**	0.218	** < 0.001**
CRP	0.937	0.207	0.748	0.103
Haematocrit (%)	0.196	** < 0.001**	** < 0.001**	** < 0.001**

Significant p values are given in bold

*HbSS* homozygous pathological haemoglobin; *HbSC* double heterozygous pathological haemoglobin; *HbAS and HbAC* asymptomatic form; *HbAA* normal haemoglobin; *F* female; *M* mal; *CRP C* Reactiv protein; *SD* standard deviation; *µL* microlitre, *IQR* interquartile range, *G* gram, *mL* millilitre

**Table 2:** Distribution of different haemoglobin phenotypes by age group

		HbAA (n = 72)	HbAS	HbAC	HbSS	HbSC
			(n = 19)	(n = 7)	(n = 23)	(n = 13)
Mean age (years). [± SD]		12.90 ± 13	13.47 ± 10.67	12.43 ± 4.76	10.17 ± 4.42	19.15 ± 14.12
< 5 yrs	N (%) Patients	29 (40.28%)	6 (31.58%)	0 (0%)	5 (21.74%)	2 (15.38%)
	Mean age [± SD]	3.3 ± 1.44	3.33 ± 1.36	–	4.6 ± 0.55	4 ± 0.0
> 5 yrs	N (%) Patients	43 (59.72%)	13 (68.42%)	7 (100%)	18 (78.26%)	11 (84.61%)
	Mean age [± SD]	19.37 ± 13.33	18.15 ± 9.75	12.43 ± 4.76	11.72 ± 3.68	21.91 ± 13.60
*P-Values*			HbAA vs HbAS	HbAA vs HbAC	HbAA vs HbSS	HbAA vs HbSC
> 5 yrs	Mean age		0.668	0.089	0.172	0.161

*N* number of patients, *HbSS* homozygous pathological haemoglobin; *HbSC* double heterozygous pathological haemoglobin; *HbAS and HbAC* asymptomatic form; *HbAA* normal haemoglobin; *SD* standard deviation; *yrs* years

### Ring-stage survival assay (RSA) and standard maturation test

**Fig. 2:**
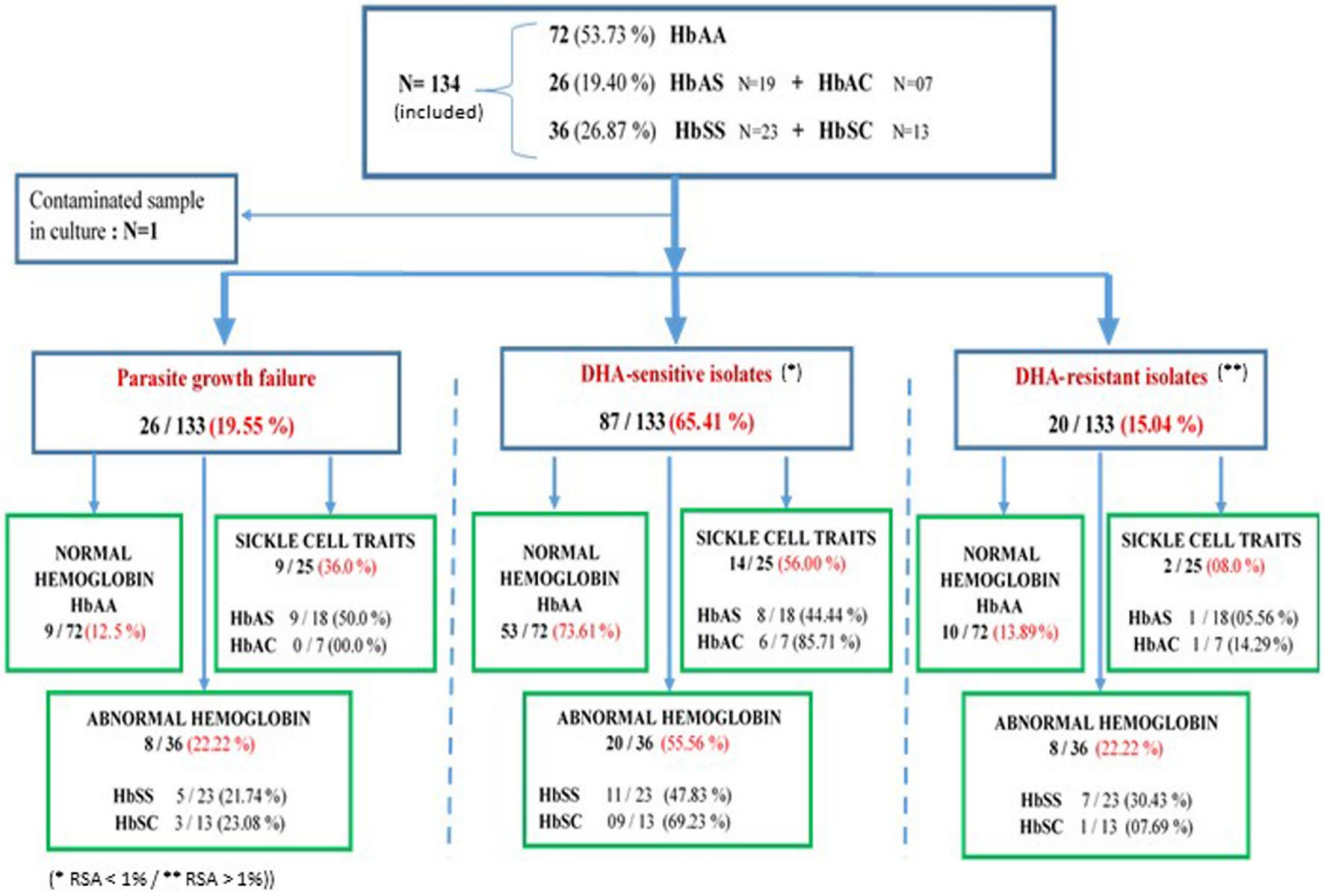
Flow chart of the results of the ex vivo RSA test 700 nM DHA

**Fig. 3:**
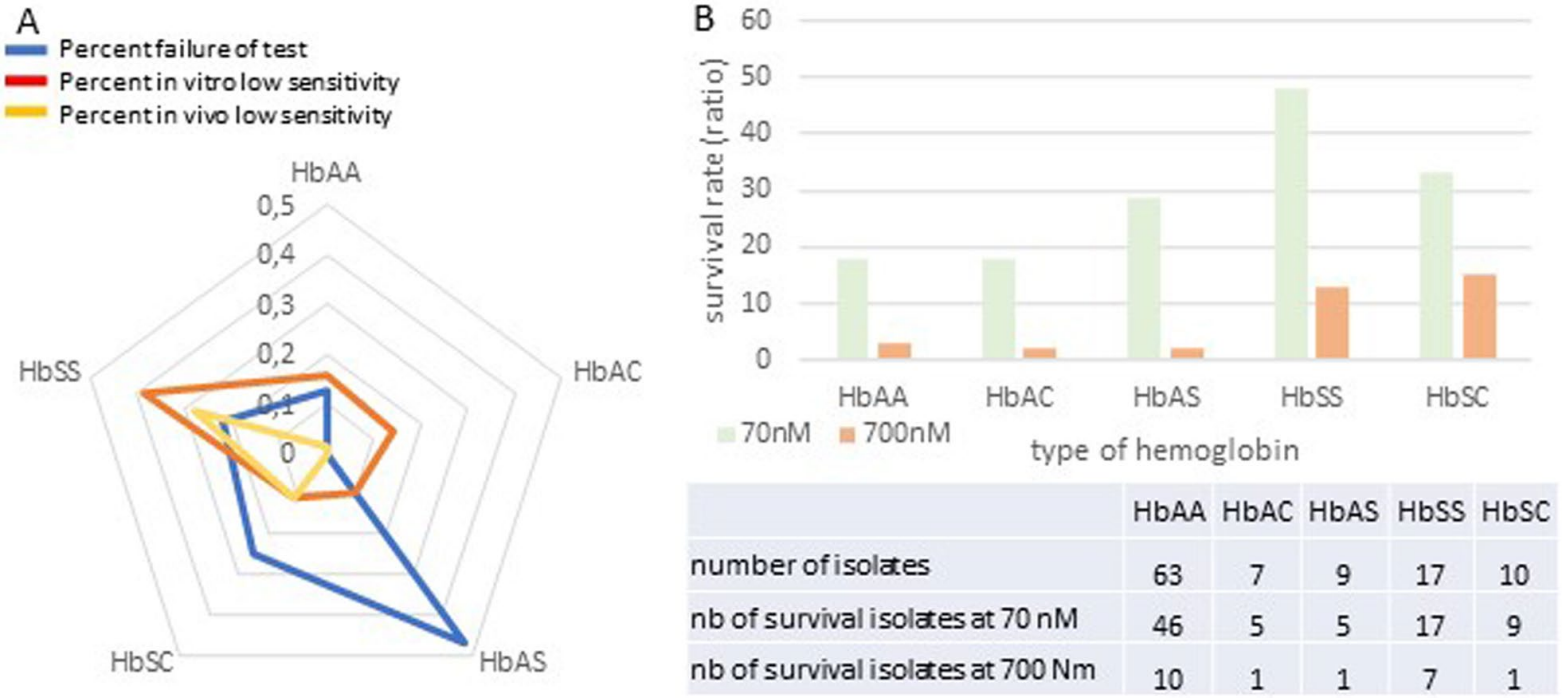
Ex vivo RSA test. **A** frequency of test failure and low-sensitivity according to the type of haemoglobin. **B** Sensitivity of isolates to DHA according to the type of haemoglobin. When the proportion of viable parasites in the non-exposed culture (DMSO) at 72 h was higher than the initial parasitaemia at 0 h, the samples were considered to be interpretable. Survival rates is the ratio of parasitaemia in exposed (DHA) and non-exposed cultures (DMSO) calculated as: (parasitaemia at 70 or 700 nM DHA exposed/parasitaemia at 0 nM control) × 100. Geometric means of the isolates is plotted. Isolates with a survival rate of more than 1% were classified as in vitro artemisinin-resistant isolates, and with more than 10% as in vivo resistant

RSA and standard maturation tests were conducted in parallel for 134 and 116 patients respectively. Only 80% (107/134) and 96% (111/116) of RSA and standard maturation test were respectively successful (Fig. 2). Indeed, a low rate of parasitic growth occurred more often with HbAS red cell (Figs. 2, 3A). RSA values for all collected clinical isolates varied from 0 to 33.75% (i.e. ratio of parasitaemia in culture with DHA 700 nM and in culture without DHA). Based on RSA results and according to a threshold of maturation rate of 1%, 65% (87/133) of the clinical isolates were sensitive (< 1%), and 15% (20/133) had a decreased of sensitivity (> 1%) to DHA 700 nM (Fig. 2B).

**Fig. 4:**
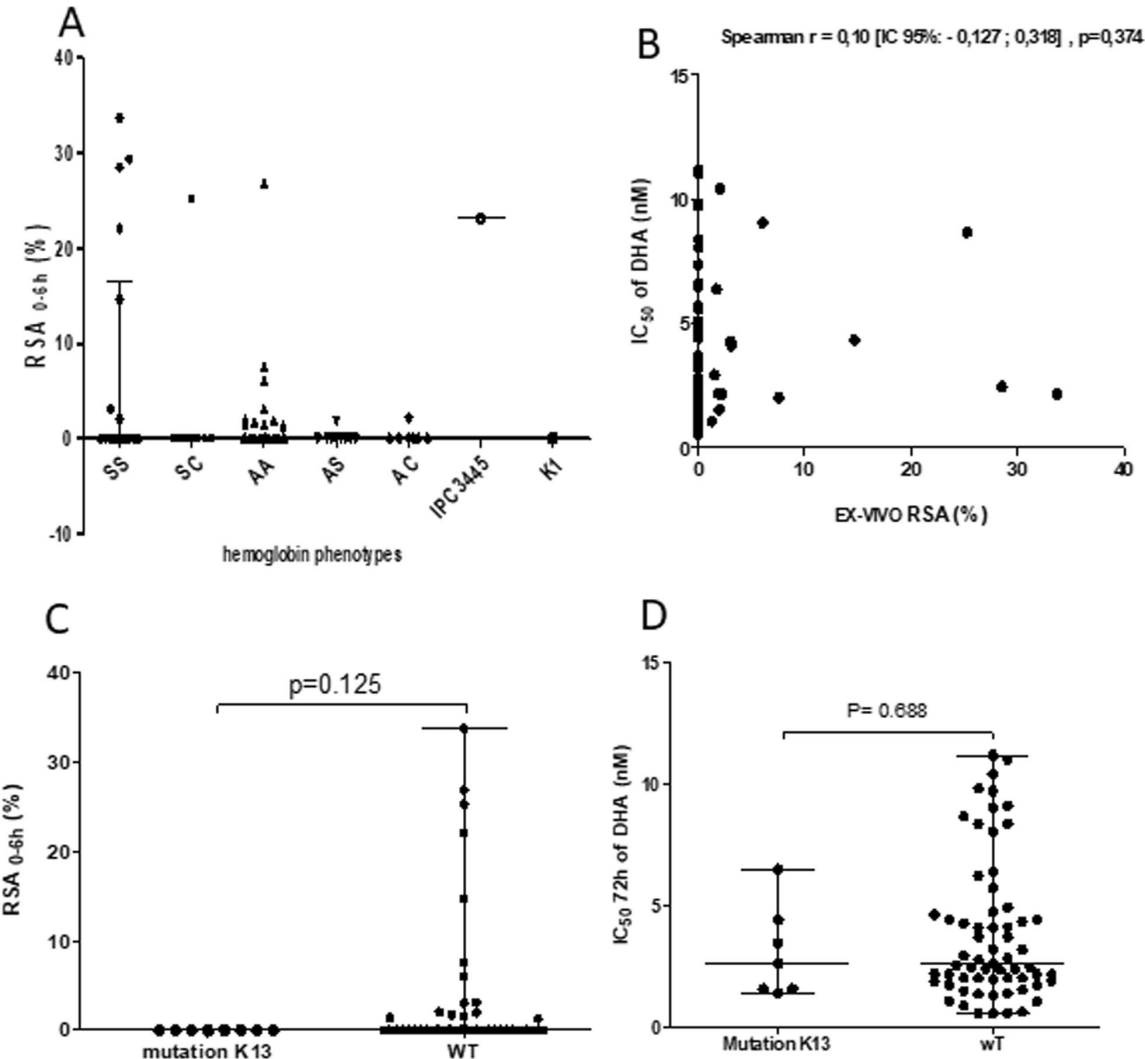
In vitro parasites drug sensitivity results. **A** Distribution of *Plasmodium falciparum* survival rates in RSA according to the type of haemoglobin. Survival rate was calculated as previously for 700 nM DHA. Geometric means with 95% confidence intervals of the survival rates are shown. **B** Spearman’s correlation between RSA (%) and IC_50_ values for DHA for the same isolate. **C** Correlation of parasite survival rates (Ex-vivo RSA) and *k13* polymorphisms. RSA values between parasites with mutations in the kelch propeller domain (> 440 amino acid) and parasites without kelch mutations. **D** Comparison of CI_50_ values for DHA between the group of *k13* wild-type parasite and the group with mutations in the propeller domain (> 440 amino acid)

For these twenty isolates, the RSA values varied from 1.28 to 33.75%. All the types of sickle-cell phenotypes were concerned by this decreased sensitivity, but 39% of them were for the HbSS group (Figs. 2, 3B). Indeed, 28% of isolates from HbSS phenotype had survival rates ranging from 14.68% to 33.75% (Figs. 3, 4A).

In these isolates, proportions of early ring-stage parasites at enrolment were high (70% to 75% of the parasites detected were rings). Parasites developed in HbSS haemoglobin are thus less sensitive in culture to DHA than parasites grown in HbAS, HbAC, HbSC and HbAA haemoglobin (Figs. 2, 3 and 4A). K1 parasite had 0% survival rate whereas IPC 3445 had 23.14%. Three clinical isolates from HbSS patients exhibited higher survival rates (28,53%, 29,40% and 33,75%) at the DHA 700 nM than the artemisinin-resistant IPC 3445 clone (Fig. 4A).

In the same way, 86% (95/111) of the in vitro standard maturation test were successful, with Inhibition Concentration 50% (IC_50_) ranging between 0.53 and 11.18 nM (geometric mean: 2.71 nM, CI [2.32–3.17], range: [0.53–11.18]) for a threshold of resistance to DHA at 10 nM. However, when comparing RSA and maturation tests with the same isolates (Fig. 4B), some isolates with a DHA-sensitive phenotype in the RSA presented a resistant phenotype in standard maturation test and vice versa. In this study, the pairwise comparison of the two tests was not significant (n = 81, Spearman r = 0.10 [95% CI − 0.127; 0.318], p = 0.374). Maturation test IC_50_ values were also not correlated with RSA survival rates.

### Relations between RSA test and clinical and biologic parameters at enrolment

In order to take into account confounding variables, quantitative parameters of hosts (age, body mass index, CRP, haemoglobin level, haematocrit) were compared firstly with parasite growth rates, and secondly between RSA susceptible or less sensitive (below and above 1%) isolates. These parameters were also compared between RSA sensitivity and partially resistance isolates (with parasite survival rate higher than 10% at 700 nM) (Table 3).

**Table 3:** Relationship between Ex vivo RSA test results and patients clinical and biologic parameters at enrolment

Count of P. f isolates	Failure of RSA test			In vitro RSA resistance test (RSA > 1%)			Significant in vivo resistance (RSA > 10%)		
	Failure of RSA test	Successful RSA test	p value	Sensitive (RSA survival < 1%)	Resistant (RSA survival ≥ 1%)	p value	Sensitive (RSA survival < 10%)	Resistant (RSA survival ≥ 10%)	p value
	N = 26	N = 107		N = 87	N = 20		N = 107	N = 07	
	mean (N)	mean (N)		mean (N)	mean (N)		mean (N)	mean (N)	
Age	13.00 (26)	11.00 (107)	0.892	11.00 (87)	12.00 (20)	0.740	11.00 (102)	13.00 (7)	0.492
ParaT0	21130 (26)	24070 (107)	0.454	30795 (87)	44778 (20)	**0.006**	22730 (102)	43900 (7)	0.076
CRP	96.00 (26)	44.00 (106)	0.151	42.00 (86)	67.50 (20)	0.843	41.00 (101)	96.00 (7)	0.524
GB	14.77 (25)	13.94 (100)	0.410	13.80 (80)	16.19 (20)	0.110	13.90 (95)	21.80 (7)	0.118
GR	03.07 (25)	04.32 (100)	**0.016**	04.37 (80)	3.81 (20)	**0.013**	04.37 (95)	01.63 (7)	**0.002**
HB	08.37 (26)	10.40 (105)	**0.005**	10.60 (85)	10.30 (20)	0.220	10.82 (100)	05.67 (7)	**0.003**
PLAQ	271.1 (25)	231.0 (100)	0.541	222.5 (80)	236.0 (20)	0.403	230.0 (95)	319.0 (7)	0.314
HCT	28.10 (25)	37.08 (100)	**0.006**	37.40 (80)	35.40 (20)	**0.009**	37.40 (95)	14.50 (7)	**0.001**
BMI	18.85 (26)	18.30 (105)	0.940	19.00 (87)	16.80 (20)	0.118	18.60 (100)	15.50 (7)	0.100
Temp	38.45 (26)	39.10 (107)	0.060	39.10 (87)	38.40 (20)	0.076	39.10 (100)	37.90 (7)	0.064

Significant p values are given in bold

N represents the number of isolates, the numbers in brackets represent the mean; RSA, ring-stage survival assay

*ParaT0*, parasitaemia at inclusion (per µL); *CRP* GB Leukocytes level (× 1000/µL); *GR* Erythrocytes level (million/µL); *HB* Haemoglobin level (G/100 mL); *PLAQ* Platelets level (× 1000/µL); *HCT* Haematocrit (%); *BMI* Body Mass Index; *Temp* temperature (°C)

Firstly red blood cell count, haemoglobin level and haematocrit level at inclusion were significantly lower in patients carrying isolates with failure of the test, compared to isolates with growth rate > 1% (Mann Whitney test, P = 0.016, P = 0.005, and P = 0.006 respectively) (Table 3). In the same way, patients with DHA- less sensitive isolates (RSA > 1% at DHA 700 nM) had a lower red blood cell count and haematocrit level compared to DHA-sensitive isolates. DHA-partially resistant isolates (RSA > 10%) had also a significantly higher parasitaemia at inclusion than DHA-sensitive isolates (44778 ± 22347 Vs 30795 ± 26671; Mann Whitney test, P = 0.006).

For HbSS patients, RSA DHA- less sensitive isolates had a significantly higher parasite growth rate compared to DHA-sensitive isolates (5.04 ± 4.92% Vs 1.57 ± 0.44%; Mann Whitney test, P = 0.0002). For the isolates with very low sensitivity (partial resistance RSA > 10%) low red blood cell count, haemoglobin level and haematocrit at inclusion were observed only for HbSS patients (P-value: 0.002, 0.003, and 0.001, respectively). Overall, parasites collected in patients with HbSS phenotype harbored more often isolates with less sensitivity to DHA 700 nM (Table 3).

### Survival rate and point mutations in the PfKelch 13 gene

Genomic DNA was obtained from 134 isolates and an 849 bp PCR fragment corresponding to the Kelch13 Propeller region was amplified and sequenced. Polymorphism analysis was possible for 74% (99/134) of these PCR products. Overall, 16 SNPs (Single Nucleotide Polymorphisms) were detected for only 7% of the sequences (i.e., 7/99). Among these SNPs, 94.44% were non-synonymous mutations (15/16). The synonymous mutation (*G287G*) was located before the propeller region of the *k13* gene (< 442 amino acids), while the 15 non-synonyms were located in this propeller region. Mutations were all different (Table 4). No key mutations already identified in the Kelch 13 propeller domain by other authors (such as *C580Y, R539T, Y493H, P574L, I543T, F446I, R561H, A675V*) and associated with a delay in parasite clearance was found. Only one mutation was found for patients with HbSS or HbAC phenotypes. No mutation was associated with a decreased drug susceptibility both for RSA and standard maturation test. No difference in IC_50_ values for DHA 700 nM was found between isolates with and without mutations (Mann Whitney U test, P = 0.688) (Fig. 4C, D).

**Table 4:** Mutations identified in *kelch13 propeller* gene of *Plasmodium falciparum* in abnormal haemoglobin (sickle cell disease) patients

Type of mutation	clinical isolates	Type of Haemoglobin	Sensitivity to DHA	Nucleic acid	Amino-Acid	HbAA	HbAS	HbAC	HbSS	HbSC
Synonymous	ANK-074	HbAA	Sensitive	GGC→GGT	G287G	1	–	–	–	–
Non-synonymous	ANK-030	HbAC	Sensitive	TTA→TTT	L462F	–	–	1	–	–
	ANK-035	HbAA	Sensitive	TGG→GGG	W565G	1	–	–	–	–
				AAT→TAT	N585Y	1	–	–	–	–
				GGT→GCT	G595A	1	–	–	–	–
				TAT→TCT	Y635S	1	–	–	–	–
				GGA→AGG	G450R	1	–	–	–	–
				AAT→AAA	N498K	1	–	–	–	–
	ANK-054	HbAA	Sensitive	GTG→GCG	V568A	1	–	–	–	–
	ANK-060	HbAA	Sensitive	AAT→CAT	N554H	1	–	–	–	–
				TAT→CAC	Y588H	1	–	–	–	–
				TTT→GTT	F451V	1	–	–	–	–
				GTG→GGT	V520G	1	–	–	–	–
	ANK-034	HbAA	Parasite growth failure	TGT→AGT	C447S	1	–	–	–	–
				TCT→TTT	S549F	1	–	–	–	–
	YOP-042	HbSS	Sensitive	TAT→GAT	Y519D	–	–	–	1	
Mutations observed	7	–		16	16	14	0	1	1	0
Total sequenced 99		–		–	–	57	13	3	17	9

Sensitivity to DHA (RSA survival ≥ 1% = ‘low sensitivity” and RSA survival < 1% = ‘Sensitive’)

## Discussion

In vitro tests and gene polymorphism analysis combined with in vivo clinical studies, can serve as predictive markers for epidemiological surveillance of artemisinin resistance [19, 52, 54]. However, very few studies address sensitivity of isolates from patients with abnormal haemoglobin. This question is of importance, as in Côte d’Ivoire almost 20% of the population is carrying at least an abnormal haemoglobin gene. Homozygote patients are at risk of severe occlusive crisis when infected with malaria. In the same time, cytosolic content of the abnormal red blood cells [55–57] can provide a specific biochemical environment susceptible to select or promote ACT decreased sensitivity in field isolates.

The participants included in the study were divided into four groups according to their sickle cell phenotypes (HbSS, HbSC, HbAS, HbAC) and HbAA as control. No significant difference in mean age was found, underlining the efficacy of the clinical management of children with SCD at the YUH [9, 58, 59]. Likewise, patients with abnormal HbSS phenotypes had lower haemoglobin content than those with HbAS, HbAC, HbSC and HbAA forms. As already reported, parasitaemia and haemoglobin levels at inclusion were lower in sickle cell patients with HbSS and HbSC than in patients with normal phenotype [60–62]. This low parasite density could be an element explaining a protective effect against severe malaria. It could be due (i) to dehydration of red blood cells which could inhibit the invasion and growth of *P. falciparum* parasites [62–64]; or (ii) to inhibition of osmotic shock in HbSS phenotype erythrocytes [65] resulting in reduced merozoite release [66, 67]. The short lifespan of sickle cell erythrocytes and the clearance of erythrocytes infected by P*. falciparum* can reduce also the parasite density. Malaria alters the red blood cells during the endo-erythrocytic phase of the development of P. falciparum and the phenomenon of sequestration of parasitized red blood cells affects the circulation and consequently provokes a vaso-occlusive crisis. Due to their particular intra-erythrocyte microenvironment [56, 57] attention must be paid to confirm viability of the parasites during the in vitro culture. A lower dose of drug i.e., 70 nM of DHA was introduced as control during the RSA test as this low dose can be tolerated by most of the parasites and give an internal control of viability of the parasites.

Witkowski et al. showed a strong correlation between ex vivo RSA survival rates at DHA 700 nM and in vivo parasite clearance half-lives in Cambodia [45], with a 89% sensitivity and 91% specificity [45]. Overall, in this study, the survival rates obtained during RSA test at DHA 700 nM, showed higher values for isolates from patients with HbSS phenotype than others. These data suggest that these isolates have a decreased sensitivity to DHA in vitro (survival rate > 1%), and potentially in vivo (survival rate > 10%). These results could be due to a higher density of ring stages (70–75%) before culture which are known to enter quiescence in the presence of DHA 700 nM [17, 68, 69]. Because many factors such as levels of host immunity and pharmacokinetics could modulate drug clinical effectiveness, further correlation of ex vivo RSA and in vivo studies is strongly required in the various malaria endemic regions with different population ethnicities and malaria ecologies.

Nevertheless, mutations in the *k13* gene associated with decrease in sensitivity to DHA (in particular the WHO-validated *C580Y, R539T, Y493H, P574L, I543T, F446I, R561H, A675V, N458Y* [70]) have not be found during this study. This absence of link between *k13* mutations and DHA sensitivity was already described elsewhere in Africa, as in Cameroon [71], Uganda [72] and even in Cambodia [73]. However, several other genes could be involved in the resistant phenotype as falcipain 2a (FP2a) a cysteine protease and haemoglobinase. Mutations in this enzyme (FP2a) reduce enzymatic activity and haemoglobin digestion, and increase the survival rate in the ring stage of *P. falciparum* [74, 75]. Mutations outside the *k13* gene could also induce compensation effect as already reported with the *Pfcrt* gene in French Guiana [76–78]. However, this study supports overall the idea that isolates from HbSS sickle cell patients, can express DHA resistant phenotype without *k13* gene polymorphism [70, 71, 79].

However, a recent work in Abidjan underlined as well a higher genetic complexity of the parasite isolates in patients with SCD or trait compared with control ones [39]. This can point out selection of a specific set of parasites entering abnormal red cells. To test this hypothesis, sequencing of the full genome of parasites collected during this study is in process. In these strains, low sensitivity could be the result of an adaptation of the parasite to novel micro-environmental or biochemical conditions [24, 57, 80] with a different transcriptome regulation and activation of specific genes [81]. Gene expression analysis studies should than be conducted. Abnormal RBCs contain could also simply inhibited artemisinin efficacy by inactivation or decrease interaction of the molecule with its target(s). Resistance could at last be explained by activation of alternative metabolic pathways as the “unfolded protein pathways” which seem up-regulated to attenuate artemisinin-induced protein damage [81]. One of the last hypotheses is a higher capacity of resistant parasite to tackle with high level of oxidative radical as these are particularly high in SCD. Resistant parasites have an undoubtable advantage to develop in these cells.

## Conclusion

This work demonstrates that malaria isolates can exhibit low DHA-sensitivity when HbSS RBCs in vitro, which is not related to polymorphism in the propeller region of the *Pfkelch 13* gene. The decreased sensitivity of *P. falciparum* to anti-malarial drugs will challenge malaria control. This study also provides evidence of an absence of relationship between *Pfkech13* polymorphism and survival rate in RSA test in sickle cell patients living in Abidjan. Taken together, these results highlight the need for appropriate and effective treatment in these subjects to protect them from severe attacks and to avoid the emergence of truly resistant strains.

## Data Availability

The data that support the findings of this study are available from Andre Toure but restrictions apply to the availability of these data, as they are not publicly available. Data are however available from the authors upon reasonable request and with permission of Ministry of Health of Ivory Coast.

## Abbreviations

ACT: Artemisinin-based combination therapy
SCD: Sickle cell disease
DHA: Dihydro-artemisinin
ROS: Reactive oxygen species
YUH: Yopougon University Hospital
ANK: Community health centre of Anonkoua-kouté
PCR: Polymerase chain reaction
FRET: Fluorescence Resonance Energy Transfer
RSA: Ring-stage survival assay
IC_50_: Inhibition Concentration 50%
CI: Confidence interval
WHO: World Health Organization
Hb: Haemoglobin
